# Clinical and radiographic comparison of sybograf® and ostin® in treating periodontal intrabony defects

**DOI:** 10.6026/973206300211860

**Published:** 2025-07-31

**Authors:** Kaushal Pati Tripathi, Rohit Mishra, Varsha Choubey, Rajul Kaurav, Anshul Gulati, Arohi Aglawe

**Affiliations:** 1 Department of Periodontology, Faculty of Dental Sciences, IMS, Banaras Hindu University, Varanasi, Uttar Pradesh, India; 2 Department of Periodontics & Implantology, Hitkarini Dental College and Hospital, Jabalpur, India

**Keywords:** Generalized chronic periodontitis, infrabony defects, Sybograf ®, Ostin ®

## Abstract

The capacity of Sybograf and Ostin to regenerate was compared in chronic periodontitis of three-wall vertical periodontitis defects. The material was randomly given for 30 patients and the clinical and radiographic assessments done at the baseline and after 3, 6, and 9 months. Sybograf (R) produced more effect of PPD reduction (4.2 mm, 72.4%) and RAL gain (4.3 mm, 32.58%) when compared to Ostin (R ) at 9 months. There was no gross discrepancy between the intergroup bone fill (Sybograf there was bone fill) and (Ostin there was bone fill) 33.1 per cent and 25.5 per cent, respectively. Sybograf 2 characteristic showed superior clinical results and both substrates were capable of regenerating bone efficiently.

## Background:

Periodontitis is derived from the terms "periodont," referring to the tissues that support the teeth, and "itis," which denotes inflammation. It encompasses a group of chronic inflammatory conditions affecting the gingiva, periodontal ligament, and alveolar bone-structures essential for maintaining tooth stability [1]. The primary objective of periodontal therapy is to arrest disease progression while promoting the regeneration of the damaged supporting structures. Periodontal regeneration involves the biological reconstruction of the cementum, alveolar bone, and periodontal ligament in areas where these tissues have been compromised [2]. Current regenerative strategies involve the use of growth factors, bone grafts, guided tissue regeneration (GTR) membranes, root surface conditioners and emerging approaches like stem cell therapy. While these methods have shown promising results, their outcomes remain somewhat unpredictable [3]. Growth factors, which are polypeptide hormones, play a crucial role in tissue repair by enhancing the production of extracellular matrix and promoting the proliferation and migration of osteoblasts, cementoblasts and periodontal ligament cells. Among the key growth factors involved in periodontal wound healing are platelet-derived growth factor (PDGF), insulin-like growth factor (IGF) and transforming growth factor-beta (TGF-β) [4]. To achieve periodontal regeneration, clinicians employ a variety of bone graft materials, including autografts, allografts, xenografts, and synthetic alternatives (alloplasts). Among the alloplastic materials used in periodontal therapy are porous and non-porous hydroxyapatite (HA), nano-hydroxyapatite, beta-tricalcium phosphate, polymethyl methacrylate, hydroxyethyl methacrylate polymers and bioactive ceramics [3]. Hydroxyapatite is particularly favored due to its excellent biocompatibility, non-toxicity, osteoconductivity and similarity to the mineral composition of natural bone [5]. Sybograf® is a synthetic nanocrystalline hydroxyapatite material available in various formulations. It is characterized by its bio resorbable, osteoconductive, non-toxic, non-pyrogenic, and hypoallergenic nature [6]. Similarly, Ostin® is composed of nanocrystalline hydroxyapatite, exhibiting calcium phosphate properties akin to those of human bone. It is known for its synthetic, biocompatible, and osteoconductive properties [7]. Given the biological potential of these materials, the present clinical study was designed to assess and compare the clinical and radiographic effectiveness of Sybograf® and Ostin® in the treatment of infrabony defects in patients with chronic generalized periodontitis. Therefore, it is of interest to compare and report the clinical and radiographic outcomes of Sybograf® and Ostin® in the management of infrabony defects in chronic generalized periodontitis.

## Materials and Methods:

The present clinical study was conducted at the Department of Periodontics and Implantology, Hitkarini Dental College and Hospital, Jabalpur (India), involving twenty patients (male and female) aged between 30 and 55 years, each diagnosed with intrabony periodontal defects. Inclusion criteria comprised patients clinically diagnosed with generalized chronic periodontitis, exhibiting probing depths of ≥5 mm along with radiographic evidence of bone loss. All subjects were in good general health and were not undergoing any systemic medication. Exclusion criteria included individuals with poor oral hygiene after phase I therapy, pregnant or lactating women, and those with systemic conditions or infectious diseases. Ethical approval for the study was obtained from the Institutional Ethics Committee, and informed consent was obtained from all participants. After initial phase I therapy, patients received maintenance care and underwent clinical evaluation four weeks post-therapy. The materials used in the study included Sybograf® and Ostin®. Sybograf® is a synthetic nanosized bioceramic hydroxyapatite powder, manufactured via biomimetic patented technology. Its nanocrystals, embedded in a silica gel matrix, measure approximately 60 nm, forming conical granules with an average length of 2 mm and diameter of 0.6 mm, having 60-80% porosity. Ostin® is a 35% nanocrystalline hydroxyapatite paste that mimics the morphology of natural bone crystals and promotes bone regeneration and healing through osteoconductivity and biocompatibility. Participants were randomly allocated into two groups using a coin toss. Group A received Sybograf® and Group B received Ostin®, both placed in the intrabony defect following full-thickness mucoperiosteal flap elevation. Clinical follow-ups were scheduled at 7 days and subsequently at 1, 3, 6, and 9 months postoperatively. Clinical parameters recorded at baseline included the Gingival Index (GI), probing pocket depth (PPD), and clinical attachment level (CAL), all measured using a UNC-15 periodontal probe with the help of a customized occlusal stent. Radiographic evaluation was performed using the long-cone paralleling technique for intraoral periapical radiographs at baseline and at 6 months. Digital images were magnified at 5X using Adobe Photoshop 7.0 and analyzed through AutoCAD 2010 software using a standardized 0.5 mm grid for linear measurements. Routine blood investigations were performed for all subjects, including hemoglobin percentage, bleeding time, clotting time, total and differential leukocyte counts, and random blood glucose levels. Serological screening for HIV and Hepatitis B was also conducted using ELISA. All surgical procedures were carried out under local anesthesia. A full-thickness flap was elevated via crevicular incisions, followed by debridement of the defect and thorough root planing. The site was then irrigated with normal saline. In Group A, Sybograf® was used to fill the defect, while Ostin® was applied in Group B. The graft material was placed incrementally from the base of the defect to the crest of the residual bone wall. Flaps were repositioned and secured with 4-0 black braided silk interrupted sutures, and surgical sites were protected with a non-eugenol periodontal dressing. Postoperative care included a prescription of Diclofenac sodium 50 mg twice daily and Amoxicillin 500 mg thrice daily for five days. Sutures and dressing were removed after one week, followed by irrigation of the surgical area with saline. All patients exhibited satisfactory healing with no adverse effects. Professional plaque control and reinforcement of oral hygiene instructions were provided at every recall visit. Final clinical and radiographic evaluations were performed at 9 months post-surgery using GI, PPD, and relative clinical attachment level assessments. Data compilation was performed using Microsoft Excel 2007 and statistical analysis was conducted using SPSS version 20. Data followed a normal distribution, allowing the use of parametric tests. Comparative analysis between Sybograf® and Ostin® groups was performed using the Student's t-test. Repeated measures ANOVA evaluated changes over time within groups, and the Mann-Whitney U-test was applied to assess intergroup differences at various time points. A p-value ≤ 0.05 was considered statistically significant.

## Results:

The clinical and radiographic assessments conducted over the 9-month follow-up period confirmed that both treatment modalities-Sybograf® and Ostin®-resulted in statistically significant improvements in clinical parameters, including Gingival Index (GI), Probing Pocket Depth (PPD), Clinical Attachment Level (CAL), and radiographic bone fill when compared to baseline measurements. At baseline, the mean plaque index for the Sybograf® group was 0.42 ± 0.13, while the Ostin® group recorded a slightly lower value of 0.34 ± 0.13. At the 3-month follow-up, both groups showed reductions in plaque accumulation with scores of 0.34 ± 0.11 for Sybograf® and 0.27 ± 0.04 for Ostin®. These values continued to decline at the 6-month mark to 0.31 ± 0.07 and 0.25 ± 0.08, respectively. The lowest mean values were observed at 9 months, with Sybograf® recording 0.21 ± 0.20 and Ostin® 0.15 ± 0.05. Although both groups exhibited progressive reductions in plaque levels over time, intergroup comparison revealed no statistically significant difference in plaque index at any evaluation point. Initial GI values were 0.51 ± 0.13 for Sybograf® and 0.48 ± 0.17 for Ostin®. At 3 months, both groups showed reductions to 0.37 ± 0.15 and 0.25 ± 0.11, respectively. Further improvement was observed at 6 months with GI scores of 0.27 ± 0.12 for the Sybograf® group and 0.17 ± 0.09 for the Ostin® group. By the 9-month follow-up, GI had reduced to 0.06 ± 0.09 in the Sybograf® group, while the Ostin® group achieved a complete resolution with a score of 0.00. Despite the evident clinical improvement in both groups, statistical analysis revealed no significant difference between them. At baseline, PPD was 7.73 ± 0.45 mm in the Sybograf® group and 7.27 ± 1.43 mm in the Ostin® group. Significant reductions were observed at 3 months, with depths decreasing to 6.67 ± 0.48 mm and 5.73 ± 1.48 mm, respectively. These improvements continued through 6 and 9 months. By the end of the study, the mean PPD in the Sybograf® group was 4.60 ± 0.50 mm, while the Ostin® group achieved a lower mean depth of 3.60 ± 0.82 mm. Intergroup comparison demonstrated a statistically significant difference in PPD reduction between the two groups, with a p-value of 0.001 (Table 1 - see PDF). Mean baseline CAL was 6.73 ± 0.45 mm for Sybograf® and 6.00 ± 1.77 mm for Ostin®. These values improved notably by the 3-month assessment, registering 5.80 ± 0.41 mm for Sybograf® and 4.67 ± 1.63 mm for Ostin®. Further gains were seen at 6 months (5.27 ± 0.45 mm vs. 3.87 ± 1.72 mm) and at the final 9-month follow-up (4.53 ± 0.83 mm for Sybograf® and 3.13 ± 1.72 mm for Ostin®). Statistical analysis confirmed that both groups experienced significant clinical attachment gain over time, with a p-value of 0.009 indicating a meaningful intergroup difference (Table 2 - see PDF). The mean percentage of bone fill at 9 months was 33.95% in the Sybograf® group and 31.1% in the Ostin® group. Although both groups demonstrated radiographic evidence of bone regeneration, there was no statistically significant difference in bone fill between them, suggesting comparable regenerative outcomes (Table 3 - see PDF and Table 4 - see PDF).

## Discussion:

The primary objective of periodontal therapy is to arrest the progression of periodontal disease and preserve the natural dentition, not only for functional mastication but also for aesthetic purposes. A critical component of treatment involves regenerating the periodontium to its original architecture after destruction caused by periodontal disease. The success of regenerative outcomes is often influenced by site-specific and patient-related factors [8]. Over the years, various regenerative modalities-including bone grafts, bone substitutes, guided tissue regeneration (GTR), and bioactive molecules-have been investigated for the management of intrabony defects in periodontitis. These approaches aim to stimulate periodontal cell proliferation, differentiation, and tissue integration [3]. Clinical studies consistently demonstrate that the application of bone grafts and substitutes results in significant improvements in probing depth reduction, clinical attachment gain and radiographic bone fill, which are indicative of effective defect healing [5]. Hydroxyapatite (HA), due to its chemical resemblance to natural bone mineral and its biocompatibility, has become a widely studied biomaterial in periodontal regenerative therapy. In particular, nanocrystalline HA exhibits enhanced biological activity, supporting osteoblast adhesion, osseointegration, and new bone formation more efficiently than microcrystalline forms [7]. In this study, two nanocrystalline hydroxyapatite-based materials-Sybograf® and Ostin®-were assessed for their regenerative potential in intrabony periodontal defects.

The clinical parameters evaluated included gingival index, probing pocket depth (PPD), and clinical attachment level (CAL), recorded at baseline, 3 months, and 9 months. Radiographic assessment using intraoral periapical radiographs (IOPA) was carried out at baseline and 9 months to determine bone fill. Since dimensional changes in periodontal tissues tend to stabilize within 9 months post-treatment, this duration was selected for follow-up. Although histological evaluation is the gold standard for assessing true periodontal regeneration, its invasive nature and ethical constraints render it unsuitable for routine clinical research. This randomized, prospective, parallel-group trial compared the effectiveness of Sybograf® and Ostin® in conjunction with open flap debridement. The results indicated that both treatment groups demonstrated statistically significant improvements in clinical and radiographic parameters when compared to baseline values and to surgical debridement alone, consistent with previously reported findings [9, 10-11]. Specifically, by 9 months, the mean PPD had reduced to 4.60 ± 0.50 mm in the Sybograf® group and 3.60 ± 0.82 mm in the Ostin® group. CAL improved to 4.53 ± 0.83 mm and 3.13 ± 1.72 mm in the respective groups. Radiographic analysis showed a mean bone fill of 33.1% in Group A (Sybograf®) and 31.94% in Group B (Ostin®). These outcomes suggest that both materials offer substantial clinical benefits, with Sybograf® showing slightly better performance in attachment level gains and bone fill, possibly due to its physical structure and biomimetic properties.

## Conclusion:

Both Sybograf® and Ostin® demonstrated significant improvements in soft tissue healing and radiographic bone fill in the treatment of intrabony defects. While Sybograf® exhibited marginally superior outcomes in probing depth and attachment gain; the results for both materials were statistically significant. Future long-term studies with larger sample sizes are recommended to validate these findings and establish the definitive role of nanocrystalline hydroxyapatite-based grafts in periodontal regeneration.
